# Trends in demographic and clinical characteristics and initiation of antiretroviral therapy among adult patients enrolling in HIV care in the Central Africa International epidemiology Database to Evaluate AIDS (CA‐IeDEA) 2004 to 2018

**DOI:** 10.1002/jia2.25672

**Published:** 2021-06-21

**Authors:** Adebola A Adedimeji, Donald R Hoover, Qiuhu Shi, Hae‐Young Kim, Ellen Brazier, Jonathan Ross, Gad Murenzi, Christella Twizere, Patricia Lelo, Dominique Nsonde, Rogers Ajeh, Anastase Dzudie, Denis Nash, Marcel Yotebieng, Kathryn Anastos, for the Central Africa IeDEA Consortium, Nimbona Pélagie, Patrick Gateretse, Jeanine Munezero, Valentin Nitereka, Théodore Niyongabo, Christelle Twizere, Hélène Bukuru, Thierry Nahimana, Elysée Baransaka, Patrice Barasukana, Eugene Kabanda, Martin Manirakiza, François Ndikumwenayo, Jérémie Biziragusenyuka, Ange Marie Michelline Munezero, Tabeyang Mbuh, Kinge Thompson Njie, Edmond Tchassem, Kien‐Atsu Tsi, Mark Benwi, Marc Lionel Ngamani, Victorine Nkome, Falone Sandjong, Akindeh Mbuh, Djenabou Amadou, Amadou Dodo Balkissou, Eric Ngassam, Eric Walter Pefura Yone, Alice Ndelle Ewanoge, Norbert Fuhngwa, Ernestine Kendowo, Chris Moki, Denis Nsame Nforniwe, Catherine Akele, Faustin Kitetele, Martine Tabala, Emile Wemakoy Okitolonda, Cherubin Ekembe, Merlin Diafouka, Martin Herbas Ekat, Adolphe Mafou, Nicole Ayinkamiye, Jules Igirimbabazi, Emmanuel Ndamijimana, Emmanuel Habarurema, Marie Luise Nyiraneza, Dorothee Mukamusana, Liliane Tuyisenge, Catherine Kankindi, Christian Shyaka, Marie Grace Ingabire, Bonheur Uwakijijwe, Jules Ndumuhire, Marie Goretti Nyirabahutu, Yvette Ndoli, Oliver Uwamahoro, Ribakare Muhayimpundu, Sabin Nsanzimana, Eric Remera, Esperance Umumararungu, Lydia Busingye, Alex M Butera, Josephine Gasana, Thierry Habiryayo, Charles Ingabire, Jules Kabahizi, Jean Chrysostome Kagimbana, Faustin Kanyabwisha, Gallican Kubwimana, Benjamin Muhoza, Athanase Munyaneza, Francoise Musabyimana, Francine Mwiza, Gallican Nshogoza Rwibasira, Jean d'Amour Sinayobye, Patrick Tuyisenge, Chantal Benekigeri, Jacqueline Musaninyange, Adebola Adedimeji, Madeline Dilorenzo, Lynn Murchison, Diane Addison, Heidi Jones, Elizabeth Kelvin, Sarah Kulkarni, Matthew Romo, Olga Tymejczyk, Batya Elul, Xiatao Cai, Allan Dong, Don Hoover, Chunshan Li, Qiuhu Shi, Robert Agler, Kathryn Lancaster, Mark Kuniholm, Andrew Edmonds, Angela Parcesepe, Jess Edwards, Olivia Keiser, Stephany Duda, April Kimmel

**Affiliations:** ^1^ Department of Epidemiology and Population Health Albert Einstein College of Medicine/Montefiore Medical Center Bronx NY USA; ^2^ Department of Statistics Rutgers University Piscataway NJ USA; ^3^ Department of Public Health School of Health Sciences and Practice New York Medical College Valhalla NY USA; ^4^ School of Public Health City University of New York New York NY USA; ^5^ Department of Medicine Albert Einstein College of Medicine Bronx NY USA; ^6^ Division of Clinical Education Rwanda Military Hospital Kanombe, Kigali Rwanda; ^7^ Centre National de Reference en Matière de VIH/SIDA Bujumbura Burundi; ^8^ Pediatric Hospital Kalembe Lembe Kinshasa Democratic Republic of Congo; ^9^ CTA Brazzaville Brazzaville Republic of Congo; ^10^ Clinical Research Education Networking and Consultancy Yaounde Cameroon

**Keywords:** ARV, Africa, cohort studies, HIV epidemiology, low‐ and middle‐income countries, HIV care continuum

## Abstract

**Introduction:**

The Central Africa International epidemiology Database to Evaluate AIDS (CA‐IeDEA) is an open observational cohort study investigating impact, progression and long‐term outcomes of HIV/AIDS among people living with HIV (PLWH) in Burundi, Cameroon, Democratic Republic of Congo (DRC), Republic of Congo (ROC) and Rwanda. We describe trends in demographic, clinical and immunological characteristics as well as antiretroviral therapy (ART) use of patients aged > 15 years entering into HIV care in the participating CA‐IeDEA site.

**Methods:**

Information on sociodemographic characteristics, height, weight, body mass index (BMI), CD4 cell count, WHO staging and ART status at entry into care from 2004 through 2018 were extracted from clinic records of patients aged > 15 years enrolling in HIV care at participating clinics in Burundi, Cameroon, DRC, ROC and Rwanda. We assessed trends in patient characteristics at enrolment in HIV care including ART initiation within the first 30 days after enrolment in care and calculated proportions, means and medians (interquartile ranges) for the main variables of interest.

**Results:**

Among 69,176 patients in the CA‐IeDEA cohort, 39% were from Rwanda, 24% from ROC, 18% from Cameroon, 14% from Burundi and 5% from DRC. More women (66%) than men enrolled in care and subsequently initiated ART. Women were also younger than men (32 vs. 38 years, *P* < 0.001) at enrolment and at ART initiation. Trends over time show increases in median CD4 cell count at enrolment from 190 cells/µL in 2004 to 334 cells/µL in 2018 at enrolment. Among those with complete data on CD4 counts (60%), women had a higher median CD4 cell count at care entry than men (229 vs. 249 cells/µL, *P* < 0.001). Trends in the proportion of patients using ART within 30 days of enrolment at the participating site show an increase from 16% in 2004 to 75% in 2018.

**Conclusions:**

Trends from 2004 to 2018 in the characteristics of patients participating in the CA‐IeDEA cohort highlight improvements at entry into care and subsequent ART initiation including after the implementation of Treat All guidelines in the participating sites.

## 1 Introduction

In 2018 over 37.9 million people aged >15 years were living with HIV globally, of whom 24.5 million (64.6%) were estimated to be accessing antiretroviral therapy (ART) [1]. Expanded access to ART contributed to improved quality of life and declines in AIDS‐related mortality in people living with HIV (PLWH) [1, 2]. To consolidate these gains and end the threat of HIV to global public health, the Joint United Nations Program on AIDS (UNAIDS) recommended the “90‐90‐90” strategy to ensure 90% of PLWH are diagnosed and know their status, 90% of those diagnosed are initiated on ART and 90% of those initiating ART attain viral suppression. To facilitate the attainment of these targets, the World Health Organization (WHO) put out the “Treat All” guidelines in 2015, which recommended immediate ART initiation for all persons diagnosed with HIV regardless of CD4 count or viral load [2].

Sub‐Saharan Africa (SSA) is still disproportionately burdened by HIV. In 2018, 73% of all new global HIV infections were in the region [3]. In most of SSA, progress towards the 90‐90‐90 targets is uneven with many countries in East and Southern Africa making remarkable progress compared with those in West and Central Africa [4, 5]. Whereas the 25 countries in West and Central Africa comprised only 6% of the world’s population, they accounted for 21% of new HIV infections and 30% of global deaths from AIDS‐related illnesses in 2018 [6]. The UNAIDS estimates that 4.7 million PLWH in West and Central Africa lack access to treatment, a sharp contrast with treatment coverage in East and Southern Africa regions [6]. These discrepancies have led to a regional catch‐up plan to accelerate progress in West and Central Africa, but this has not translated into meaningful gains in scaling up access for millions of PLWH in the region [6].

The lack of progress reflects disparities in access to care for many PLWH in Central Africa who continue to experience late diagnosis, non‐linkage to care and poor HIV outcomes [1, 2, 3, 4, 5, 6, 7]. Social and structural instability, especially stigma, weak health systems, civil unrest and other contextual factors impede access to HIV care and progress towards universal targets [5, 6, 7, 8]. To address gaps in access to care for PLWH in Central Africa, it is important to understand the characteristics of PLWH enrolling in care and subsequently initiating ART. Additionally, knowledge of trends in demographic, immunological and clinical characteristics among PLWH enrolled in care can highlight challenges in implementing effective strategies to facilitate early diagnosis and linkage to care for PLWH in these countries.

The Central Africa‐IeDEA (CA‐IeDEA) research consortium, one of seven regional cohorts within the International Epidemiology Database to Evaluate AIDS (IeDEA) (https://www.iedea.org), is funded by the United States National Institutes of Health to be a resource to investigate the impact, progression and long‐term outcomes of the HIV/AIDS epidemic in Central Africa and answer epidemiologic research questions that cannot be answered by separate individual cohorts. The overall IeDEA cohort has been previously described and consists of 1.7 million PLWH of whom 1.4 million live in SSA [9, 10].

The CA‐IeDEA routinely collects sociodemographic, clinical and immunologic data from the medical records of patients enrolled in HIV care at participating treatment sites in Burundi, Cameroon, Democratic Republic of the Congo (DRC), Republic of Congo (ROC) and Rwanda. In Cameroon, some additional clinical information is collected directly from the participants. These data provide insights on trends in sociodemographic, clinical and immunological characteristics of adult patients entering HIV care and initiating ART at the participating sites before and after the introduction of the Treat All guidelines. The objective of this study was to examine these trends in sociodemographic, clinical and immunological characteristics of adult patients aged >15 years at entry into HIV care in their current sites and subsequently initiating ART in CA‐IeDEA from 2004 to 2018 with the goal of describing trends in these characteristics over time.

## 2 Methods

### 2.1 Study settings

The CA‐IeDEA cohort currently is comprised of 19 active sites in Burundi, Cameroon, DRC, ROC and Rwanda, and one historical site in DRC that contributed patient data to the cohort until mid‐2013. The 19 active sites are predominantly public sector health facilities, including tertiary‐level university teaching hospitals and primary level health centres. All the sites are located in urban or peri‐urban settings. Details of CA‐IeDEA sites have been previously described [9]. Patients ever enrolling in HIV care at these participating sites are prospectively included in the CA‐IeDEA cohort. Data from routine patient care are regularly extracted from patient records and electronic medical records and harmonized into a regional dataset for use in country‐level and regional analyses. As of July 2018, there were approximately 70,000 patients in the CA‐IeDEA database.

### 2.2 Study design and population

This analysis included all adult patients aged >15 years enrolled for HIV care in the CA‐IeDEA cohort at participating sites in Burundi, Cameroon, DRC, ROC and Rwanda. Data obtained during routine patient care in each country are extracted from an electronic data collection and storage system in each country, for example the Open Medical Records System (in Rwanda), SANTIA (https://santia.org/) (in RoC), Syndrome d’immunodeficience acquise (SIDA Info‐ http://svtedu.free.fr/log/sida.htm) (in Burundi) and RedCap (in Cameroon and DRC) as mandated by the local health authority in each country. These data are then uploaded to a central data server at the CA‐IeDEA data centre in New York where they undergo cleaning and harmonization prior to analysis.

### 2.3 Ethical approval

Ethical approval for the study was granted by the Albert Einstein College of Medicine Institutional Review Board in New York, and the relevant ethics review boards in Rwanda (Rwanda National Health Research Committee and the National Ethics Committee), Burundi (Comite National d’Ethique), Cameroon (Comite National D’Ethique la Recherche en la Sante Humaine‐ CNERSH) DRC (Ministere de l’Enseignement Superieur et Universitaire, University de Kinshasa Ecole de Sante Publique) and in the ROC (Comite d’Ethique De La Recherche En Sciences De La Sante (CERRSSA). Data for the Central Africa International Epidemiology to Evaluate AIDS (CA‐IeDEA) are available upon request.

As per ethical review board guidelines in Burundi, DRC, ROC and Rwanda, a consent waiver was obtained as study staff did not initiate any contact with patients whose data were included in this analysis. By design, only de‐identified secondary data were extracted from routine clinical care data as approved by the various ethics review boards. The only exception was in Cameroon, where informed written consent is obtained from patients actively enrolled in HIV care at study sites before conducting structured interviews with these patients or abstracting data from medical records.

### 2.4 Measures

The following sociodemographic characteristics were assessed at entry into care at the participating CA‐IeDEA site: age, gender, marital status, reported pregnancy among women and route of entry into HIV care in the participating site such as voluntary counselling and testing (VCT), prevention of mother‐to‐child transmission (PMTCT), Tuberculosis (TB) clinic or In‐patient care. Clinical and immunological characteristics assessed at entry into HIV care at the participating CA‐IeDEA site included weight, height, body mass index (BMI), CD4 cell count, WHO stage at enrolment and ART use. ART use prior to enrolment was defined as ART use >30 days before entering care in the participating site. ART use immediately after entering HIV care at the participating site was defined as ART initiation starting ≤30 days before enrolment to 30 days after enrolment in HIV care at the participating site. We considered CD4 cell count measures at enrolment as the available CD4 measure closest to the enrolment date 30 days prior or up to three months after enrolment. CD4 cell count measure at ART initiation was defined as the first CD4 measure available 30 days prior and up to three months after ART initiation date among patients who initiated ART use after enrolment.

### 2.5 Statistical analysis

For this analysis, we calculated proportions and medians (interquartile ranges) for the main variables of interest. We ultimately categorized all continuous variables as shown in the Tables and used Chi‐Square tests to make the comparison of all variables between groups. We also calculated the same data summaries by calendar year of enrolment into HIV care at the CA‐IeDEA participating site and by country. Overall and within country descriptive presentation and comparisons of the measures of interest described in the previous paragraph were made stratified by sex and/or use of ART (categorized as prior to study entry versus initiation within 30 days of care entry). Trends of these measures at enrolment into care and ART initiation at enrolment over calendar time both overall and within country were plotted on Figures.

## 3 Results

### 3.1 Demographic and clinical profile of all adults at enrolment into care

The demographic and clinical profile of 69,176 patients entering HIV care in the CA‐IeDEA cohort in all the five participating countries is shown in Table 1. The largest proportion of patients enrolled in care at the participating sites was in Rwanda (39%) and the smallest proportion (5%) in the DRC. Almost twice as many women (66%) as men enrolled in HIV care at participating sites. The median age at enrolment was 35 years (IQR: 28, 42); women were generally younger (33 years; IQR: 27, 40) than men (38 years; IQR: 32, 45). The median CD4 count at entry into care was 282 cells/µL (IQR: 139, 476) among those for whom the CD4 cell count measure was available. Women enrolled in care at the CA‐IeDEA sites with higher CD4 count (299 cells/µL, IQR: 151, 501) than men (249 cells/µL; IQR: 119, 427). About 46% of patients who enrolled in care at the participating sites did not use any ART up to three months after enrolment; 11% initiated ART prior to (>30 days) enrolment in HIV care at the CA‐IeDEA site – likely patients transferring from another site where they were already initiated on ART – and 42% initiated ART within 30 days (+/−) of enrolment in HIV care.

**Table 1:** Demographic and clinical profile of all adult patients at enrolment into care by sex

Variable	Code	Total	Women	Men
		69176	45933 (66.4)	23243 (33.6)
Pregnant			2869 (6.2)	
Age (years) median (IRQ)	Median (IQR)	35 (28,42)	33 (27,40)	38 (32,45)
	15 to 24	8894 (12.9%)	7291 (15.9%)	1603 (6.9%)
	25 to 34	25623 (37.0%)	19088 (41.6%)	6535 (28.1%)
	35 to 44	22031 (31.8%)	13113 (28.5%)	8918 (38.4%)
	45+	12628 (18.3%)	6441 (14.0%)	6187 (26.6%)
Point of entry into care	PMTCT	6530 (9.4%)	5623 (12.2%)	907 (3.9%)
	Tuberculosis Clinic	38 (0.1%)	27 (0.1%)	11 (0.0%)
	VCT	21285 (30.8%)	12372 (26.9%)	8913 (38.3%)
	Inpatient	1009 (1.5%)	524 (1.1%)	485 (2.1%)
	Other	6177 (8.9%)	3904 (8.5%)	2273 (9.8%)
	Unknown	34137 (49.3%)	23483 (51.1%)	10654 (45.8%)
Marital status	Single	15894 (23.0%)	10911 (23.8%)	4983 (21.4%)
	Married/in union	19345 (28.0%)	10735 (23.4%)	8610 (37.0%)
	Living with partner	3413 (4.9%)	2372 (5.2%)	1041 (4.5%)
	Divorced	3153 (4.6%)	2326 (5.1%)	827 (3.6%)
	Widowed	5073 (7.3%)	4299 (9.4%)	774 (3.3%)
	Unknown/Missing	22298 (32.2%)	15290 (33.3%)	7008 (30.2%)
Weight for adults, kg	Median (IQR)	57 (50,65)	56(49,64)	59 (53,67)
	Missing n (%)	20702 (29.9%)	13901 (30.3%)	6801 (29.3%)
Height for adults, cm	Median (IQR)	163 (158,169)	160 (155,165)	170 (165,175)
	Missing n (%)	20555 (29.7%)	13551 (29.5%)	7004(30.1%)
Body mass index (kg/m^2^)	Median (IQR)	21.63 (19.27,24.38)	22.07 (19.53,24.98)	20.94 (18.93,23.31)
	Missing n (%)	31156 (45.0%)	20702 (45.1%)	10454 (45.0%)
CD4 count (cells/µL)	Median (IQR)	282 (139,476)	299 (151,501)	249 (119,427)
	<100	7500 (10.8%)	4610 (10.0%)	2890 (12.4%)
	100 to 199	7513 (10.9%)	4846 (10.6%)	2667 (11.5%)
	200 to 349	10093 (14.6%)	6739 (14.7%)	3354 (14.4%)
	>350	16456 (23.8%)	11811 (25.7%)	4645 (20.0%)
	Missing (%)	27614 (39.9%)	17927 (39.0%)	9687 (41.7%)
WHO stage at enrolment	Stage I	21004 (30.4%)	14247 (31.0%)	6757 (29.1%)
	Stage II	12961 (18.7%)	8659 (18.9%)	4302 (18.5%)
	Stage III	11167 (16.1%)	6967 (15.2%)	4200 (18.1%)
	Stage IV	3698 (5.3%)	2257 (4.9%)	1441 (6.2%)
	Missing n (%)	20346 (29.4%)	13803 (30.1%)	6543 (28.2%)
Country	Rwanda	26930 (38.9%)	16651 (36.3%)	10279 (44.2%)
	Burundi	9720 (14.1%)	6409 (14.0%)	3311 (14.2%)
	Cameroon	12482 (18.0%)	8323 (18.1%)	4159 (17.9%)
	DRC	3292 (4.8%)	2718 (5.9%)	574 (2.5%)
	ROC	16752 (24.2%)	11832 (25.8%)	4920 (21.2%)
Time period	Year 2006 or prior	15054 (21.8%)	10246 (22.3%)	4808 (20.7%)
	Year 2007 to 2010	17488 (25.3%)	11717 (25.5%)	5771 (24.8%)
	Year 2011 to 2014	17761 (25.7%)	12125 (26.4%)	5636 (24.2%)
	Year >= 2015	18873 (27.3%)	11845 (25.8%)	7028 (30.2%)
ART Status at enrolment	Not on ART	32233 (46.6%)	21357 (46.5%)	10876 (46.8%)
	Before enrolment^a^	7601 (11.0%)	5268 (11.5%)	2333 (10.0%)
	Immediately after enrolment^b^	29342 (42.4%)	19308 (42.0%)	10034 (43.2%)

^a^ >30 days prior to enrolment day

^b^ within 30 days after enrolment day.

### 3.2 Characteristics of patients initiating ART within 30 days of enrolling in HIV care and patients initiating ART prior to enrolment in care in CA‐IeDEA sites

Table 2 shows the characteristics of all patients who initiated ART within 30 days of enrolment in care and patients who were already using ART when they enrolled in care in the participating site. HIV Voluntary Counselling and Testing (HIV‐VCT) was the source of entry into care for 25% of those who initiated ART at enrolment in care and 38% of those already using ART prior to enrolling in care at the current site.

**Table 2:** Characteristics of patients starting ART within 30 days of enrolment and those already using ART before enrolment in care

Variable	Code	Start ART within 30 days of enrolment	Using ART before enrolment
Adult		29342	7601
Pregnant		NA	NA
Age (years) median (IQR)	Median (IQR)	35 (29,43)	36 (30,43)
	15 to 24	3402 (11.6%)	750 (9.9%)
	25 to 34	10324 (35.2%)	2568 (33.8%)
	35 to 44	9654 (32.9%)	2605 (34.3%)
	45+	5962 (20.3%)	1678 (22.1%)
Point of entry into care	PMTCT	2748 (9.4%)	840 (11.1%)
	Tuberculosis Clinic	7 (0%)	0 (0.0%)
	VCT	7219 (24.6%)	2903 (38.2%)
	Inpatient	434 (1.5%)	98 (1.3%)
	Other	2342 (8.0%)	716 (9.4%)
	Unknown	16592 (56.5%)	3044 (40.0%)
Marital status	Single	8427 (28.7%)	1174 (15.4%)
	Married/in union	9388 (32.0%)	1588 (20.9%)
	Living with partner	2145 (7.3%)	374 (4.9%)
	Divorced	1538 (5.2%)	339 (4.5%)
	Widowed	2669 (9.1%)	650 (8.6%)
	Unknown/missing	5175 (17.6%)	3476 (45.7%)
Weight for adults, kg	Median (IQR)	58 (50,65)	59 (52,67)
	Missing n (%)	6254 (21.3%)	1228 (16.2%)
Height for adults, cm	Median (IQR)	164 (158,170)	162 (157,169)
	Missing n (%)	4943 (16.8%)	1311 (17.2%)
Body mass index (kg/m^2^)	Median (IQR)	21.6 (19.10, 24.34)	22.22 (19.92, 25.0)
	Missing n (%)	9905 (33.8%)	2379 (31.3%)
CD4 count (cells/µL)	Median (IQR)	203 (100, 340)	361 (205, 543)
	<100	4686 (16.0%)	549 (7.2%)
	100 to 199	4575 (15.6%)	684 (9.0%)
	200 to 349	5160 (17.6%)	1241 (16.3%)
	>350	4418 (15.1%)	2653 (34.9%)
	Missing	10503 (35.8%)	2474 (32.5%)
WHO stage at enrolment	Stage I	9171 (31.3%)	2064 (27.2%)
	Stage II	6348 (21.6%)	1238 (16.3%)
	Stage III	6021 (20.5%)	1650 (21.7%)
	Stage IV	1935 (6.6%)	465 (6.1%)
	Missing	5867 (20.0%)	2184 (28.7%)
Country	Rwanda	9091 (31.0%)	5159 (67.9%)
	Burundi	3900 (13.3%)	290 (3.8%)
	Cameroon	9371 (31.9%)	1849 (24.3%)
	DRC	1046 (3.6%)	165 (2.2%)
	ROC	5934 (20.2%)	138 (1.8%)
Time period	Year ≤2006	3540 (12.1%)	484 (6.4%)
	Year 2007 to 2010	5395 (18.4%)	1603 (21.1%)
	Year 2011 to 2014	7618 (26.0%)	2566 (33.8%)
	Year ≥2015	12789 (43.6%)	2948 (38.8%)

IQR, interquartile range; PMTCT, prevention of mother‐to‐child transmission; VCT, voluntary counselling and testing.

Overall, there were important similarities and differences regarding clinical characteristics between those who initiated ART within 30 days of enrolment and those who were already using ART prior to enrolment in care. While the median weight, height, BMI and WHO stage were similar among the two groups, the median CD4 cell count was lower among patients initiating ART at enrolment in care (203 cell/µL; IQR: 100, 340) vs. patients already using ART prior to enrolling in care at the participating site (361 cell/µL; IQR: 205, 543). Nearly two‐thirds of patients in Rwanda were already using ART prior to enrolling in care at the participating clinic, the most of any country.

We also observed differences between women and men who initiated ART within 30 days of enrolment and those already using ART prior to enrolling in care at the current site. For example women make up the majority of patients who initiated ART at enrolment in care or prior to enrolment in care at the participating clinic. Women also tend to be younger than men among those initiating ART at enrolment in care (34 years IQR: 28, 41 vs. 39 years IQR: 32, 45), *P* < 0.001 and among those already using ART prior to enrolling in care (35 years, IQR: 29, 41 vs. 40 years, IQR: 33, 47), *P* < 0.001 in the participating site. The median CD4 cell count was also higher among women who initiated ART at enrolment in care (216 cells/µL, IQR: 104,356) vs. men (182 cells/µL, IQR: 82,309), *P* < 0.001 and among women already using ART (392 cells/µL, IQR: 232, 584) vs. men (293 cells/µL, IQR: 160, 454), *P* < 0.001.

### 3.3 Trends in the median CD4 cell count, ART use prior to and at enrolment in care and WHO stage among patients in all participating clinics

Patients’ CD4 cell count and WHO stage are important clinical parameters that indicate the overall state of health of PLWH before and after enrolment in care. Before the WHO’s 2015 Treat All recommendations, CD4 cell count was an important criterion for determining eligibility for ART initiation. While CD4 testing is no longer required for determining treatment eligibility, pretreatment CD4 testing is still recommended in order to identify patients with advanced disease who are at elevated risk for opportunistic illness and mortality.

**Figure 1:**
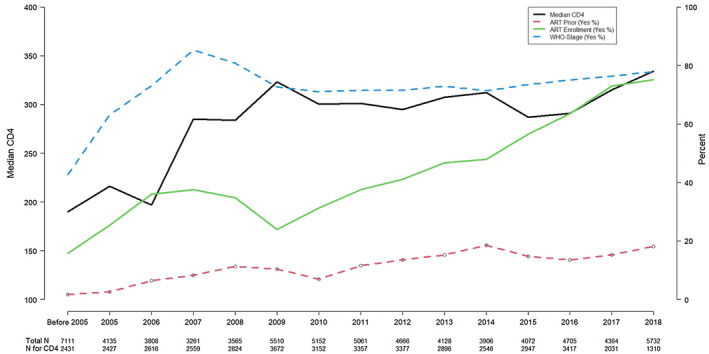
Trends in the median CD4 cell count, proportion of patients with ART use prior to and at enrolment in care, and available WHO stage in all participating countries.

Figure 1 shows yearly trends in the median CD4 cell count at enrolment, ART use among patients prior to enrolment in care, ART initiation among patients when they enrolled in care and WHO stage for all patients in the participating sites. These yearly trends are based on the availability of data for the variable included. The median CD4 cell count for all patients when they enrolled in care increased from 190 cells/µL in the period before 2005 to 334 cells/µL in 2018. Before 2005, the proportion of respondents using ART before they enrolled in HIV care at the current CA‐IeDEA site was less than 2% but this proportion increased to 18% by 2018. There was also a progressive increase in the proportion of patients who started using ART when they enrolled in care in all the years except in 2009. The proportion of patients with recorded WHO stage also increased sharply from 2005 to 2007 before falling slightly and levelling off. The proportion of patients initiating ART within 30 days of enrolment grew from 16% in 2005 to 75% in 2018. It is worth noting that these trends generally remained high after the Treat All guideline was implemented in the CA‐IeDEA participating countries. Country‐specific data on the median CD4 cell count and ART use before and after enrolment in HIV care for all patients are shown in Figures S1‐S10.

## 4 Discussion

In this study describing trends in demographic, clinical and immunological characteristics of the largest cohort of adult PLWH entering care and initiating ART in Central Africa from 2004 to 2018, we found increases in baseline CD4 cell count among patients enrolling in HIV care in the participating CA‐IeDEA sites and in the proportion of first time ART users within 30 days of entry into care at participating sites in Burundi, Cameroon, DRC, ROC and Rwanda. Despite these increases, in all five countries, men still have lower CD4 counts than women at enrolment in care and at ART initiation, indicating that they lag behind in entering HIV care and initiating treatment.

These findings highlight the progress and challenges in early HIV diagnosis and ART initiation that are critical to achieving the universal goal of eliminating the virus by 2030. Equally important, our findings highlight the differences in demographic and clinical parameters between men and women at the time of enrolment in HIV care‐ a proxy for timely HIV diagnosis and how this can determine long‐term HIV outcomes in populations of PLWH across the 5 countries in the CA‐IeDEA. Furthermore, knowledge of the characteristics between PLWH newly initiating care and those already in care and using ART can inform effective programmes and/or policies to improve access to care, long‐term clinical outcomes and quality of life and substantially reduce morbidity and mortality among PLWH in Central Africa in the era of universal ART use [11] especially for men who enrol later in care and have higher mortality rate compared to women [12, 13].

Women comprised nearly two‐thirds of patients who enrolled in care and who initiated ART within 30 days of enrolment at a CA‐IeDEA site. HIV prevalence in SSA is higher among women than men [2, 13] and women are more likely to be tested for HIV earlier than men, in part because of contacts with antenatal care during pregnancy [13]. The higher proportion of women in the CA‐IeDEA cohort may partly reflect women’s greater likelihood to access testing and care, and may also reflect a higher risk of transmission in women and perhaps higher mortality rates in men [14].

Before the WHO’s 2015 recommendation of universal HIV treatment for all PLWH, also known as “Treat All,” CD4 cell count and WHO staging were the most commonly used criteria for determining treatment eligibility [13]. Our findings suggest that at that time information on CD4 cell count and WHO staging was missing for a substantial proportion of patients, with likely implications for delayed timing of ART initiation and higher mortality [15]. Missing CD4 cell count and WHO stage data are perhaps a reflection of different contextual factors, for instance changes in recommended guidelines related to clinical care, health facility capacity to obtain these measures and other sociopolitical and economic challenges. In addition, in some countries, patients are expected to bear the cost associated with obtaining CD4 counts, which are often prohibitive given their low socioeconomic circumstances. While there were differences among countries and between women and men, the low median CD4 cell counts at entry into care at participating CA‐IeDEA sites suggest that PLWH were being diagnosed late and thus were likely entering care with advanced disease, particularly in the pre‐Treat All era. In the post Treat All era, rising the median CD4 cell counts suggests that patients were entering HIV care earlier, although it is worth highlighting that even after CD4 eligibility thresholds were eliminated, the median CD4 cell count at entry into care remained below 500 cells/µL, the treatment eligibility threshold prior to Treat All adoption. However, the large proportion of missing CD4 counts makes interpretation difficult. Clinicians may be more likely to obtain a CD4 count in patients with symptoms and signs of advanced disease, thus biasing median CD4 count levels among those with CD4 count testing measures. We also observed sex differences across countries with women having higher CD4 cell counts than men at enrolment into HIV care at the participating CA‐IeDEA sites and ART initiation, in part reflecting women’s earlier access to HIV testing and diagnosis through antenatal care in PMTCT clinics.

The fact that the median CD4 count was quite low in the pre‐Treat All era suggests that patients were not accessing treatment early enough and may have had significant immune compromise by the time treatment was initiated. Analysis of the median CD4 count by country shows that patients in Rwanda had the highest median CD4 cell count when they enrolled in care in our participating clinics, which is higher than patients in Burundi, Cameroon, DRC and ROC. The CD4 cell count in Rwanda may have disproportionately impacted the reported median CD4 cell count across the participating countries. Arguably, Rwanda’s early response to the epidemic manifested in early diagnosis and investment in the continuum of care has impacted these findings.

Over the time period of this study, there were changes in recommended criteria for ART initiation among PLWH, culminating in the Treat All guidelines recommending ART initiation immediately upon HIV diagnosis, regardless of clinical and immunological parameters [16]. These changes in treatment guidelines likely drove the increased proportion of those initiating ART use immediately after entry into care. Our findings show that ART initiation increased dramatically for PLWH enrolled from 2015 onwards, coinciding with the existence/implementation of the Treat All guidelines.

HIV VCT is an important gateway to knowing one’s HIV status. VCT is beneficial for individuals by ensuring they know their HIV status and are able to take preventive actions against HIV acquisition or transmission to others. Additionally, knowledge of HIV seropositive status increases the likelihood of early engagement in care, facilitates timely ART initiation and confers protective benefits on the entire population [17]. Consistent with evidence from other studies [18, 19], we found VCT clinics to be an important route of entry into HIV care for a large proportion of patients in our cohort and this may have contributed to the rapid initiation of ART within 30 days of enrolment.

This descriptive study utilized data from five countries to describe the demographic, clinical and immunological characteristics of PLWH in Burundi, Cameroon, DRC, ROC and Rwanda at entry into care. This large combined data set over a 14‐year period in the largest cohort of adult PLWH entering care and initiating ART in Central Africa is a strength of this study. Nonetheless, there are limitations, which warrant caution in interpretation of the findings reported here. First, we highlight the different sample sizes of the cohort in each participating country. That nearly 4 in 10 CA‐IeDEA patients were from Rwanda may have skewed the results. Second, missing data on weight, height, CD4 cell count and WHO staging in many clinics impacted the findings. Additionally, the selected clinics in largely urban and peri‐urban settings is another limitation as they do not necessarily represent all health facilities, especially those in rural settings providing HIV care in all countries. In addition, the participating clinics may be better resourced to scale up patient enrolment in care and treatment initiation, which may not reflect the disparity between urban/peri‐urban and rural health facilities.

Despite these limitations, the CA‐IeDEA cohort offers an opportunity for further longitudinal analysis of HIV and non‐communicable disease clinical outcomes, as well as the sociocontextual and health system factors that facilitate or hinder timely HIV diagnosis, enrolment in care, ART initiation among PLWH in the region. This is critical because Central Africa continues to lag behind other regions in curtailing the spread of HIV. For example in‐depth analyses are needed to examine trends in long‐term outcomes among PLWH in this region so as to generate evidence to inform programmes and policies that address the circumstances unique to each country and the region.

## 5 Conclusions

Trends in demographic, clinical and immunological characteristics of patients entering into HIV care and subsequently initiating ART in the participating sites in Burundi, Cameroon, DRC, ROC and Rwanda highlight improvements over time among those accessing care in this cohort. Although progress is being made especially with improvements in CD4 cell count and the number of patients initiating ART soon after enrolling in care, men continue to enter care and initiate ART later than women in these countries. Our findings support previous reports that gaps still remain in scaling up access to care for men living with HIV in the region. Therefore, additional research is needed, including long‐term follow‐up in the CA‐IeDEA cohort, to better understand the reasons men do not access timely care and to inform the implementation of contextually relevant programme and policy strategies to increase access to timely HIV care and improving long‐term outcomes.

## Competing interests

The authors have no competing interest to declare.

## Authors’ contributions

AA wrote initial drafts and revised the manuscript. AA, QS, DH, HYK, JR, GM, MY and KA. performed research, curated data, performed analysis and contributed to initial drafts and revisions. EB, CT, PL, DMN, RA, AD and DN contributed to initial drafts and read revised drafts of the manuscript. All authors have read and approved the manuscript.

## Supporting information


**Figure S1.** Trends in proportions having a recent measure for BMI, CD4 cell count and WHO stage in the Burundi CA‐IeDEA cohort
**Figure S2.** Trends in proportions having a recent measure for BMI, CD4 cell count and WHO stage in the Cameroon CA‐IeDEA cohort
**Figure S3.** Trends in proportions having a recent measure for BMI, CD4 cell count and WHO stage in the DRC CA‐IeDEA cohort
**Figure S4.** Trends in proportions having a recent measure for BMI, CD4 cell count and WHO stage in the ROC CA‐IeDEA cohort
**Figure S5.** Trends in having a recent measure for BMI, CD4 cell count and WHO stage at enrollment in HIV care in the Rwanda CA‐IeDEA cohort
**Figure S6.** Trends in CD4 cell count and ART use before enrollment and after enrollment in HIV care among PLWH in Burundi CA‐IeDEA cohort
**Figure S7.** Trends in CD4 cell count and ART use before enrollment and after enrollment in HIV care among PLWH in Cameroon CA‐IeDEA cohort
**Figure S8.** Trends in CD4 cell count and ART use before enrollment and after enrollment in HIV care among PLWH in DRC CA‐IeDEA cohort
**Figure S9.** Trends in CD4 cell count and ART use before enrollment and after enrollment in HIV care among PLWH in ROC CA‐IeDEA cohort
**Figure S10.** Trends in CD4 cell count and ART use before enrollment and after enrollment in HIV care among PLWH in Rwanda CA‐IeDEA cohort
