# Supplementary material for: Trends in demographic and clinical characteristics and initiation of antiretroviral therapy among adult patients enrolling in HIV care in the Central Africa International epidemiology Database to Evaluate AIDS (CA‐IeDEA) 2004 to 2018
Source: J Int AIDS Soc. 2021 Jun 21;24(6):e25672. doi: 10.1002/jia2.25672 (PMC8216247; doi:10.1002/jia2.25672)
Supplement: Supplementary file 1 — Figure S1. Trends in proportions having a recent measure for BMI, CD4 cell count and WHO stage in the Burundi CA‐IeDEA cohort Figure S2. Trends in proportions having a recent measure for BMI, CD4 cell count and WHO stage in the Cameroon CA‐IeDEA cohort Figure S3. Trends in proportions having a recent measure for BMI, CD4 cell count and WHO stage in the DRC CA‐IeDEA cohort Figure S4. Trends in proportions having a recent measure for BMI, CD4 cell count and WHO stage in the ROC CA‐IeDEA cohort Figure S5. Trends in having a recent measure for BMI, CD4 cell count and WHO stage at enrollment in HIV care in the Rwanda CA‐IeDEA cohort Figure S6. Trends in CD4 cell count and ART use before enrollment and after enrollment in HIV care among PLWH in Burundi CA‐IeDEA cohort Figure S7. Trends in CD4 cell count and ART use before enrollment and after enrollment in HIV care among PLWH in Cameroon CA‐IeDEA cohort Figure S8. Trends in CD4 cell count and ART use before enrollment and after enrollment in HIV care among PLWH in DRC CA‐IeDEA cohort Figure S9. Trends in CD4 cell count and ART use before enrollment and after enrollment in HIV care among PLWH in ROC CA‐IeDEA cohort Figure S10. Trends in CD4 cell count and ART use before enrollment and after enrollment in HIV care among PLWH in Rwanda CA‐IeDEA cohort [file JIA2-24-e25672-s001.docx]

**Supplementary Materials**

**Figure. S1**: **Trends in proportions having a recent measure for BMI, CD4 cell count and WHO stage in the Burundi CA-IeDEA cohort**

**Figure. S2**: **Trends in proportions having a recent measure for BMI, CD4 cell count and WHO stage in the Cameroon CA-IeDEA cohort**

**Figure S3**: **Trends in proportions having a recent measure for BMI, CD4 cell count and WHO stage in the DRC CA-IeDEA cohort**

**Fig. S4: Trends in proportions having a recent measure for BMI, CD4 cell count and WHO stage in the ROC CA-IeDEA cohort**

**Fig. S5: Trends in having a recent measure for BMI, CD4 cell count and WHO stage at enrollment in HIV care in the Rwanda CA-IeDEA cohort**

**Fig. S6:** **Trends in CD4 cell count and ART use before enrollment and after enrollment in HIV care among PLWH in Burundi CA-IeDEA cohort**

**Fig. S7:** **Trends in CD4 cell count and ART use before enrollment and after enrollment in HIV care among PLWH in Cameroon CA-IeDEA cohort**

**Fig. S8:** **Trends in CD4 cell count and ART use before enrollment and after enrollment in HIV care among PLWH in DRC CA-IeDEA cohort**

**Fig. S9: Trends in CD4 cell count and ART use before enrollment and after enrollment in HIV care among PLWH in ROC CA-IeDEA cohort**

**Fig. S10:** **Trends in CD4 cell count and ART use before enrollment and after enrollment in HIV care among PLWH in Rwanda CA-IeDEA cohort**
